# Bringing Cardiovascular Prevention into the Community: Protocol for an Observational Study of a Mosque-Based Health Initiative Combining Education and Cardiovascular Screening

**DOI:** 10.3390/jcdd13100525

**Published:** 2026-10-19

**Authors:** Nimrat Grewal, Oumaima Mohammadi, Danza Scharink, Aya Mohammadi, Manar Mohammadi, Josephine Tan, Abby Geerlings, Sabrina Oussaada, Ali Kaplan, Badr Friguech, Hiba El Hri, Simran Grewal

**Affiliations:** 1 Department of Cardiothoracic Surgery, Amsterdam University Medical Centers, Location AMC, 1105 AZ Amsterdam, The Netherlands; o.mohammadi@amsterdamumc.nl (O.M.); m.d.scharink@amsterdamumc.nl (D.S.); j.m.e.tan@amsterdamumc.nl (J.T.); a.kaplan@amsterdamumc.nl (A.K.); 2 Department of Cardiothoracic Surgery, Yale School of Medicine, New Haven, CT 06510, USA; 3 Hogeschool van Amsterdam, 1091 GH Amsterdam, The Netherlands; aya.mohammadi@hotmail.com; 4 Amsterdam University Medical Centers, Location VUmc, 1081 HV Amsterdam, The Netherlands; m.mohammadi@amsterdamumc.nl; 5 Department of Cardiology, Amsterdam University Medical Centers, 1105 AZ Amsterdam, The Netherlands; a.e.geerlings@amsterdamumc.nl (A.G.); h.elhri@amsterdamumc.nl (H.E.H.); 6 Department of Internal Medicine, Amsterdam University Medical Centers, 1105 AZ Amsterdam, The Netherlands; s.m.oussaada@amsterdamumc.nl; 7 Department of Cardiothoracic Surgery, Haga Ziekenhuis, 2545 AA The Hague, The Netherlands; bfriguech@gmail.com; 8 Department of Orthopaedic Surgery, Onze Lieve Vrouwe Gasthuis, 1091 AC Amsterdam, The Netherlands; sgrewal@jvg.nl

**Keywords:** cardiovascular prevention, community health, inverse care law, health inequalities, mosque-based interventions, cardiovascular screening, underserved populations

## Abstract

Background: Populations with the highest cardiovascular risk are often the least likely to take part in preventive care, a pattern described by the inverse care law. Delivering prevention in trusted community settings may improve reach. Methods: This protocol describes an observational, cross-sectional study embedded in recurring health days at a large mosque in Amsterdam, the Netherlands. Each health day combines a collective educational session, dialogue with healthcare professionals and a low-barrier health check by Hartcheckpunten of the Dutch Heart Foundation (blood pressure, heart rate, capillary cholesterol and glucose, as well as waist circumference), followed by lifestyle advice on elevated risk factors. Adult visitors are invited to complete a short questionnaire and, with separate consent, to allow the use of their health check results. The primary outcomes are health perception, access to healthcare, trust in healthcare and health literacy. Secondary outcomes are screen-positive cardiovascular risk factors, defined with prespecified cut-off values and distinguished from a clinical diagnosis; their relation to the primary outcomes; and feasibility, assessed with RE-AIM indicators. Analyses are descriptive, with 95% confidence intervals. Discussion: This study will show whom mosque-based health days reach, what they detect and whether they can be delivered and evaluated in this setting. It is not designed to assess effectiveness. The findings will inform the design of future community-based prevention studies.

## 1. Introduction

Cardiovascular disease remains the leading cause of mortality worldwide despite considerable advances in prevention, diagnosis and treatment. During recent decades, increasing emphasis has been placed on early identification of cardiovascular risk factors through screening programs, preventive care pathways and public health campaigns [1].

Nevertheless, improvements in cardiovascular prevention have not benefited all populations equally [2].

In Europe, the burden of cardiovascular disease and its risk factors differs considerably between migrant groups and host populations [3]. European guidelines recommend systematic cardiovascular risk assessment in adults with major vascular risk factors [4], and the 2024 ESC guidelines emphasize the detection of elevated blood pressure [5]. Both depend on contact with healthcare services.

Many existing preventive strategies are inherently healthcare-centered. Individuals are expected to engage with primary care, respond to screening invitations, attend consultations and actively seek healthcare information. While effective for many populations, such approaches rely on assumptions regarding healthcare accessibility and engagement that may not apply universally.

Participation in preventive healthcare is influenced by a broad range of factors extending beyond biological risk alone. Health literacy, migration background, language barriers, cultural perceptions of disease, competing social priorities and trust in healthcare systems may all influence healthcare utilization and preventive behavior [6,7,8]. More recent work has described the social determinants of cardiovascular disease [9], the role of health literacy in its primary prevention [10], the importance of accessible, acceptable and trustworthy healthcare for migrants in Europe [11], and the need to distinguish trust, mistrust and distrust when working towards health equity [12].

As a result, populations carrying a disproportionate burden of cardiovascular disease may paradoxically be among those least likely to participate in traditional preventive pathways.

This observation reflects the principle described by Tudor Hart in the inverse care law, which states that the availability of good medical care tends to vary inversely with the need for it within a population [13]. Although initially proposed decades ago, this concept remains highly relevant within preventive medicine and healthcare inequalities research [14].

Individuals at highest risk frequently experience barriers making engagement with preventive healthcare more difficult. Consequently, prevention programs may unintentionally reinforce existing inequalities by preferentially reaching populations already connected to healthcare systems [15].

Community-based prevention strategies increasingly seek to reposition healthcare within existing social ecosystems. Rather than asking communities to enter healthcare institutions, these approaches attempt to bring healthcare directly into trusted environments where people already gather and interact [16].

Religious institutions and community organizations may provide particularly valuable settings because they function not only as physical locations but also as social infrastructures characterized by trust, familiarity and established networks [17].

Mosques occupy a unique position within many communities. Beyond their religious role, they frequently function as places of social interaction, education and intergenerational exchange. Their role as community hubs may therefore create opportunities for health engagement that differ substantially from conventional healthcare settings [18].

Previous faith-based interventions have demonstrated promising findings in areas including diabetes education, hypertension awareness and participation in preventive care initiatives [19,20,21]. However, evidence regarding integrated community-based cardiovascular prevention programs combining education, healthcare interaction and direct cardiovascular screening remains limited. Recently, a scoping review mapped faith-driven health promotion interventions delivered in mosques [16], and a systematic review and meta-analysis evaluated cardiovascular and stroke prevention programs for culturally and linguistically diverse communities [22].

Importantly, screening itself should not be considered an isolated intervention. Identification of elevated blood pressure, cholesterol or glucose values alone does not necessarily translate into meaningful health impact. Understanding, trust and contextual relevance likely determine whether risk identification ultimately results in behavioral change or healthcare engagement [23].

We therefore developed a community-embedded cardiovascular prevention initiative hosted within a mosque setting. This paper describes the protocol of an observational study embedded in the recurring health days of this initiative.

## 2. Materials and Methods

### 2.1. Study Design

**Figure 1:**
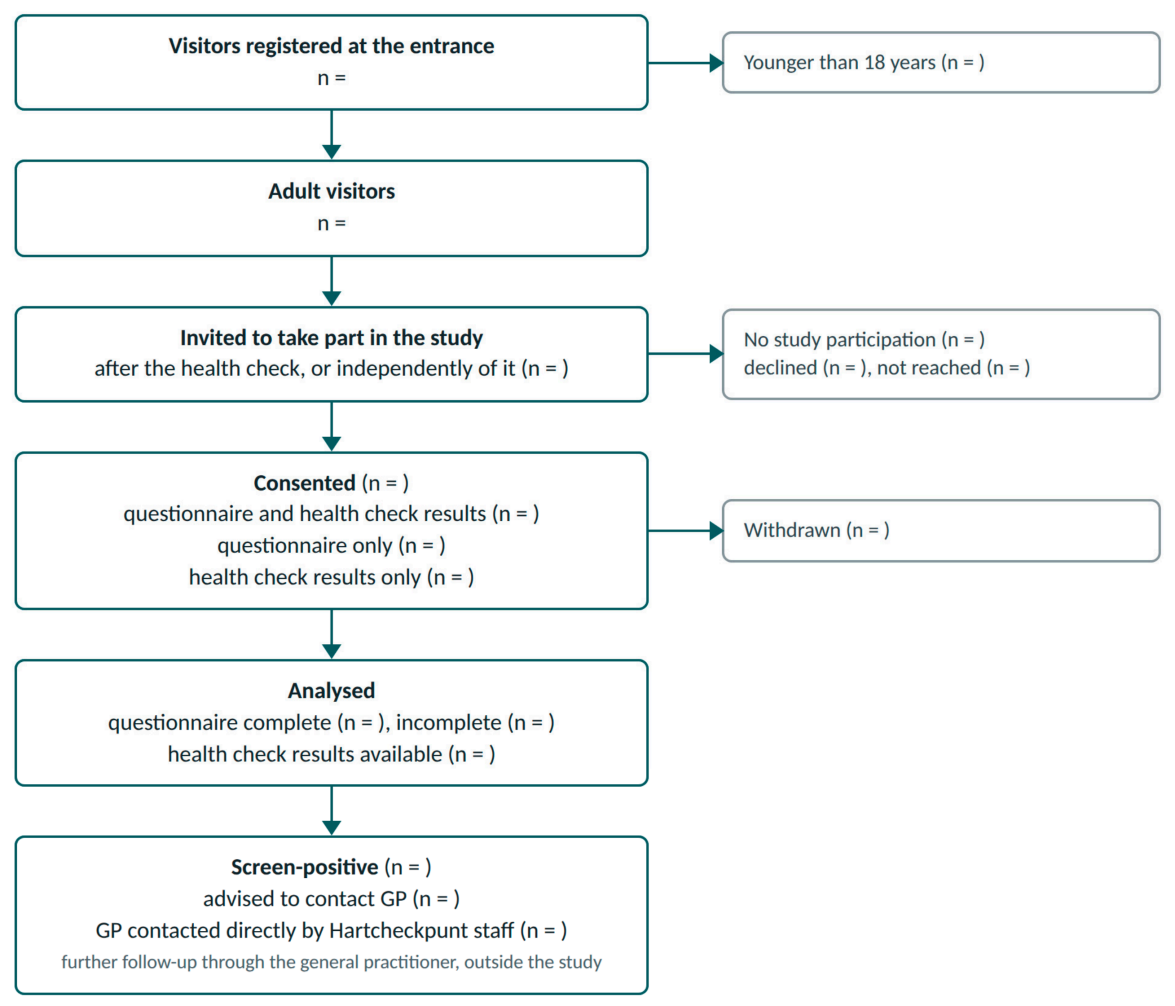
Participant flow diagram. GP, general practitioner.

This is an observational, cross-sectional study embedded in recurring community health days held at Mosque Al-Kabir in Amsterdam, the Netherlands. The health day is organized independently of this study and is open to all visitors. It consists of a collective educational session, informal dialogue with healthcare professionals and a health check at a Hartcheckpunt of the Dutch Heart Foundation. The health check is performed entirely by staff of the Dutch Heart Foundation, according to its standard procedures, and medical follow-up of abnormal values runs through the Dutch Heart Foundation and the general practitioner. The study procedures (information, informed consent and questionnaire) are carried out separately from the health check. Visitors can take part in the health check, in this study, or in both. This study consists of a short questionnaire and, with separate consent, the coded use of the health check results. The flow of participants is shown in Figure 1.

The primary objective is to describe health perception, access to healthcare, trust in healthcare and health literacy among visitors to mosque-based health days. Secondary objectives are to describe the proportion of participants with screen-positive cardiovascular risk factors; to explore the relation between these risk factors and health perception, access to care and health literacy; and to assess the feasibility of delivering and evaluating health days in mosques. As this study has no comparator, it is not designed to assess the effect of the health day on risk factor control, health behavior or clinical outcomes. The protocol follows the SPIRIT 2025 guideline [24], adapted to an observational design, and results will be reported according to STROBE [25].

### 2.2. Study Setting

The health days are held at Mosque Al-Kabir in Amsterdam, one of the largest mosques in the city, which serves a large and diverse community. The first health day has taken place, and three to four health days are planned per year at the same location. Rather than functioning as isolated screening activities, the health days are intended to establish a recurring infrastructure for community engagement and preventive healthcare in a familiar and accessible environment.

### 2.3. Study Population

All visitors aged 18 years or older are eligible. There is no further selection. Visitors who are unable to provide informed consent are not included. The questionnaire is open to all adult visitors; the health check is offered by the Hartcheckpunt to visitors aged 40 to 70 years without known cardiovascular disease and without medication for blood pressure, cholesterol or glucose (Section 2.7.3). Bilingual team members and interpreters are present at each health day, and the questionnaire is available in Dutch, Turkish, Moroccan Arabic, English, Spanish, Hindi and Punjabi. Participation is voluntary and no financial incentives are provided. Participation in the health day does not depend on participation in this study (Table 1).

### 2.4. Ethical Considerations

This study is conducted in accordance with the Declaration of Helsinki. This study was reviewed and approved by the non-WMO review committee of the Medical Ethics Review Committee of Amsterdam UMC, which confirmed that it is not subject to the Medical Research Involving Human Subjects Act (WMO) (reference 2026.0388). Participants receive written information and provide written informed consent after completing the health check, or independently of it if they take part in the questionnaire only. Separate consent is asked for the questionnaire, for the use of health check results and for storage of data for 10 years for future research. Participants can withdraw at any time without giving a reason.

### 2.5. Partnership with the Mosque and Community Engagement

The health day is organized by TRAIN Health Awareness and the Thoracic Aortic Research Group (TARGet) of Amsterdam UMC, in collaboration with the board of the mosque and the Dutch Heart Foundation, which provides the Hartcheckpunt. The collaboration with Mosque Al-Kabir was established through an existing relationship between a member of the research team and the mosque board. The mosque provides the venue and volunteers, and the health day is scheduled on a Friday directly after Friday prayer, when attendance is highest. The imam is informed two weeks in advance and announces the health day during prayers. The value of a healthy life within Islamic teaching is addressed in the Friday sermon. Volunteers of the mosque register visitors at the entrance. Visitors are also recruited through the social media channels and WhatsApp groups of the mosque.

### 2.6. Patient and Public Involvement

This protocol describes a planned community-embedded initiative in which patient and public involvement is integral to both its design and its conduct. The program is developed in close collaboration with local community organizations and mosque representatives, who help shape the choice of setting and the design, delivery and cultural relevance of the health events. Community partners will advise on the content and wording of the needs-assessment questionnaire and on the outcomes most relevant to the community, and will be involved in interpreting the findings. The results will be shared with participating communities in accessible, culturally appropriate formats, and community feedback will be used to refine and sustain future iterations of the initiative. Participants take part on a voluntary basis and are not involved in the day-to-day conduct of data collection.

### 2.7. Components of the Health Day

**Figure 2:**
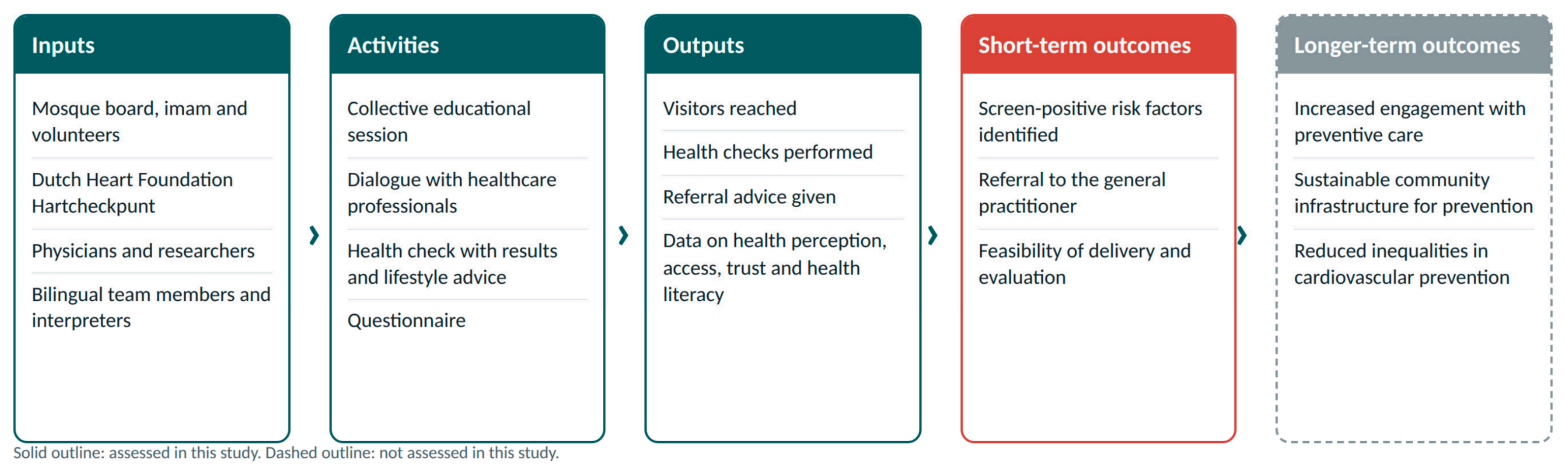
Logic model of the health day and this study. Arrows indicate the assumed sequence from inputs to outcomes. Solid outline: assessed in this study. Dashed outline (grey): not assessed in this study. The short-term outcomes (red) are the outcomes assessed in this study.

The health day consists of three components, which are described according to the TIDieR template [26] in Table 2. The logic model of the health day and this study is shown in Figure 2.

#### 2.7.1. Structure of the Health Day and Health Education

The health day takes place on a Friday afternoon, directly after Friday prayer. It starts with a collective educational session, followed by the health check. Participants receive their results and a brief explanation from the Hartcheckpunt staff. At the request of the Dutch Heart Foundation, lifestyle coaches are present to discuss the findings. Together with members of the TRAIN team, they provide general information and lifestyle advice on risk factors found to be elevated, in a conversation of approximately 10 to 15 min; individual results are not interpreted medically in this conversation. The collective session lasts approximately 45 min and is given by physicians from the research team (cardiothoracic surgery, cardiology and internal medicine) in Dutch, with interpretation by bilingual team members and interpreters. It consists of a short presentation followed by questions and discussion. The presentations consist of short slide presentations prepared by the research team, which are available from the corresponding author on request. Topics include:

- Hypertension;
- Diabetes mellitus;
- Obesity;
- Cholesterol;
- Smoking;
- Nutrition;
- Physical activity;
- Sleep;
- Stress;
- Family history and hereditary cardiovascular risk.

Cultural adaptation consists of the choice of setting and timing, the involvement of the imam, language support and the use of examples related to commonly used foods, family life and fasting during Ramadan.

#### 2.7.2. Dialogue with Healthcare Professionals

Throughout the day, visitors can engage in informal conversations with physicians, researchers and other healthcare professionals. These conversations are low-barrier opportunities for dialogue rather than structured medical consultations and cover lifestyle, cardiovascular risk, use of healthcare and prevention.

#### 2.7.3. Health Check

The health check is performed by trained staff of the Hartcheckpunt according to the standard procedures of the Dutch Heart Foundation. Blood pressure and heart rate are measured twice with an automated device; if the blood pressure is elevated, it is measured again [27]. Total cholesterol, LDL cholesterol, HDL cholesterol, non-HDL cholesterol, triglycerides and glucose are measured in capillary blood obtained by finger prick with a point-of-care device. Measurements are performed in the non-fasting state. Waist circumference, body weight and height are measured, and body mass index (BMI) is calculated. In line with the criteria of the Hartcheckpunt, the health check is offered to visitors aged 40 to 70 years without known cardiovascular disease and without medication for blood pressure, cholesterol or glucose [27]. The Hartcheckpunt staff share the results with the participant and provide a brief explanation; the subsequent lifestyle advice is described in Section 2.7.1.

#### 2.7.4. Markedly Abnormal Results

Abnormal results are handled by the Hartcheckpunt staff according to the protocols of the Dutch Heart Foundation, which were agreed upon with general practitioners [27]. Participants with abnormal values are advised to contact their general practitioner. A systolic blood pressure of 180 mmHg or higher, a diastolic blood pressure of 110 mmHg or higher or a glucose level of 15.0 mmol/L or higher requires contact with the general practitioner on the same day; for a glucose level of 11.1 to 14.9 mmol/L, the general practitioner is informed on the same or the next day. In these cases, the Hartcheckpunt staff contact the participant’s general practitioner by telephone, and the general practitioner takes over further care. Physicians are present at every health day and can provide immediate assistance in case of acute symptoms.

### 2.8. Questionnaire

Participants complete a short questionnaire (approximately 5 min; Supplementary File S1) covering the following domains:

- Background characteristics: age category, sex, country of birth (the Netherlands or elsewhere) and duration of residence in the Netherlands.
- Access to care: ease of making an appointment with the general practitioner; forgone care in the past and the reasons (language problems, lack of trust, shame, lack of time, not knowing where to go, other).
- Trust and experience: trust in Dutch healthcare; feeling understood by the physician.
- Health and lifestyle: perceived healthiness of one’s own lifestyle; topics participants want to learn more about; previous blood pressure measurement.
- Health literacy: three items adapted from the Set of Brief Screening Questions [28,29]: need for help reading information from the doctor or pharmacy, confidence in completing medical forms and difficulty understanding written health information. Limited health literacy is defined as needing help at least sometimes, having difficulty at least sometimes, or being at most somewhat confident in completing forms.
- Community and preferences: willingness to receive health information in the mosque, preferred channel (including via the imam) and willingness to take part in a health check.

These variables are used to describe which groups the health days reach; this study does not assume that visitors have limited access to healthcare.

### 2.9. Study Outcomes

The outcomes, their definitions, measurement and time points are summarized in Table 3.

#### 2.9.1. Primary Outcomes

The primary outcomes are derived from the questionnaire: health perception (perceived healthiness of own lifestyle); access to healthcare (ease of making an appointment with the general practitioner, forgone care and the reasons for it); trust in Dutch healthcare and feeling understood by the physician; and health literacy, categorized as adequate or limited (Section 2.8).

#### 2.9.2. Secondary Outcomes

Secondary outcomes are screen-positive cardiovascular risk factors; the relation between these findings and the primary outcomes; feasibility (Section 2.9.3); and preferences for health information (topics, setting and channel). Referral advice is recorded as described in Section 2.10. Screen-positive findings are defined according to the cut-off values of the Hartcheckpunten [27]:

- Elevated blood pressure: systolic 140 mmHg or higher and/or diastolic 90 mmHg or higher; markedly elevated: systolic 180 mmHg or higher and/or diastolic 110 mmHg or higher.
- Elevated glucose (non-fasting, capillary): 7.8 mmol/L or higher, reported as mildly elevated (7.8 to 11.0 mmol/L), elevated (11.1 to 14.9 mmol/L) or markedly elevated (15.0 mmol/L or higher).
- Elevated cholesterol: one or more lipid fractions classified as elevated or markedly elevated by the Hartcheckpunt. Total cholesterol is additionally reported in the categories of the Dutch Heart Foundation: below 5.0 mmol/L normal, 5.0 to 6.5 mmol/L slightly elevated, 6.5 to 8.0 mmol/L elevated and 8.0 mmol/L or higher markedly elevated.
- Increased waist circumference: 94 cm or more in men and 80 cm or more in women (increased risk); 102 cm or more in men and 88 cm or more in women (high risk).
- Obesity: BMI of 30 kg/m^2^ or higher; overweight: BMI of 25.0 to 29.9 kg/m^2^.

A single community-based measurement does not establish a diagnosis. Screen-positive findings are therefore reported as such, and participants with a screen-positive finding are advised by the Hartcheckpunt staff to have the finding assessed by their general practitioner. As the health check is only offered to visitors without known cardiovascular disease and without medication for blood pressure, cholesterol or glucose, screen-positive findings concern individuals who are not treated for the respective risk factor. As previous diagnoses are not recorded in the questionnaire, a screen-positive finding cannot formally be classified as previously unidentified; a screen-positive blood pressure is, however, additionally reported for participants who report never having had their blood pressure measured.

#### 2.9.3. Feasibility

Feasibility is assessed with the following indicators, structured according to the RE-AIM framework [30]:

- Reach: number of visitors registered at the entrance; proportion of adult visitors who take part in the health check; proportion who consent to the questionnaire; proportion of health check participants who consent to the use of their results.
- Implementation: proportion of participants with complete health check measurements; proportion who complete the questionnaire (at least 80% of items answered); missing data per item; proportion of participants who take part in the educational session, the dialogue and the health check; proportion of screen-positive participants who receive referral advice; number of staff and volunteers per health day; use of language support.
- Adoption and maintenance: number of health days held; number of partners and volunteers involved; willingness of the mosque and partners to host future health days.

Effectiveness, the remaining RE-AIM dimension, is not assessed because this study has no comparator.

### 2.10. Referral

Participants with markedly abnormal values are handled according to the procedures of the Dutch Heart Foundation (Section 2.7.4). For each participant who consents to the use of health check results, the referral advice is recorded: no referral, advice to contact the general practitioner or direct contact with the general practitioner by the Hartcheckpunt staff. Further follow-up of abnormal results takes place through the general practitioner, according to the procedure of the Dutch Heart Foundation. The research team is not involved in this care pathway, and follow-up after the health day is not part of this study, in line with its observational design.

### 2.11. Data Management and Quality Control

Questionnaires are completed on paper and, together with the health check results of participants who consented to their use, are coded and entered into a database on secure Amsterdam UMC servers. No directly identifying personal data are stored with the research data. The key linking codes to consent forms is stored in a secure location at Amsterdam UMC, and only members of the research team have access to the coded data. If a participant withdraws during or directly after completing the questionnaire, the questionnaire is destroyed and not used. Data are stored for 10 years in accordance with the applicable guidelines and the General Data Protection Regulation. Data entry is checked with built-in range checks, a random sample of 10% of the paper questionnaires is checked against the database, and missing data are reviewed after each health day so that procedures can be adjusted.

### 2.12. Sample Size

As this study is descriptive, no formal power calculation was performed. The sample size is pragmatic. Three to four health days are planned per year, each attended by approximately 400 to 500 visitors. Based on attendance and the voluntary nature of participation, we expect 200 to 400 participants per health day. The first analysis will include all participants of the health days held in the first year of this study, with an expected minimum of 600 participants. With 600 participants, a proportion of 30% is estimated with a 95% confidence interval of 26.4% to 33.8%; with 200 participants, the interval is 24.0% to 36.6% (Supplementary Table S3). This precision is sufficient to describe the study population and to inform the design of subsequent studies.

### 2.13. Statistical Analysis

Analyses follow this prespecified plan; no analyses had been performed at the time of submission of this protocol. Participant characteristics, questionnaire responses and screening results are summarized descriptively. The distribution of continuous variables is assessed with histograms and Q-Q plots; normally distributed variables are reported as means ± standard deviations and other continuous variables as medians with interquartile ranges. Categorical variables are reported as numbers and percentages.

All proportions for primary and secondary outcomes are reported with 95% confidence intervals calculated with the Jeffreys method [31]. The denominator is the number of participants with a valid response or measurement for the respective outcome. Feasibility indicators are reported with the denominators defined in Section 2.9.3.

The relation between health perception, access to care and health literacy on the one hand and screen-positive risk factors on the other is explored with chi-square or Fisher’s exact tests, *t*-tests or their non-parametric equivalents. If the number of participants with a screen-positive finding allows at least 10 events per variable, exploratory logistic regression is used, adjusted for age category and sex.

Missing data are reported per variable. Given the descriptive aim, analyses are performed on available data without imputation. Characteristics of participants with and without a complete questionnaire are compared.

Prespecified exploratory subgroup analyses: sex; age category; born in the Netherlands versus born elsewhere and duration of residence in the Netherlands; and health literacy (adequate versus limited). All exploratory analyses are hypothesis-generating; no correction for multiple testing is applied and results are interpreted accordingly. Two-sided *p*-values below 0.05 are considered statistically significant.

Analyses are performed with IBM SPSS Statistics version 32 (IBM Corp., Armonk, NY, USA) and R version 4.6.1 (R Foundation for Statistical Computing, Vienna, Austria).

### 2.14. Study Timeline

This study was reviewed by the non-WMO review committee of Amsterdam UMC (reference 2026.0388). The first health day has taken place at Mosque Al-Kabir, and three to four health days are planned per year at the same location. Data collection for the first analysis ends after the health days of the first study year; analysis and reporting follow within 6 months. No analyses had been performed at the time of submission of this protocol. The schedule of enrollment and assessments is shown in Table 4. As this is an observational study without assignment of participants to an intervention, it was not registered in a trial registry; publication of this protocol makes the prespecified design and analysis plan publicly available.

## 3. Discussion

Cardiovascular prevention strategies have traditionally been organized around existing healthcare structures, where individuals are expected to actively engage with healthcare systems through primary care consultations, screening programs and preventive services. While such approaches have improved outcomes for many populations, they may insufficiently address disparities in access and participation among underserved communities [6,7,8,9,32].

The present protocol was developed from the observation that individuals carrying a disproportionate burden of cardiovascular disease may simultaneously face greater barriers to preventive healthcare participation. This phenomenon reflects the principle of the inverse care law, whereby those with the greatest healthcare needs often experience the lowest access to healthcare services [13]. Although originally described more than five decades ago, this principle remains highly relevant within contemporary healthcare systems and preventive medicine [14].

Current cardiovascular prevention programs may unintentionally reinforce existing inequalities by predominantly reaching populations already connected to healthcare systems. Individuals experiencing barriers related to migration background, health literacy, language, socioeconomic circumstances or trust in healthcare institutions may therefore remain structurally underrepresented within conventional prevention pathways [6,7,8,11,15].

The American Heart Association has called for the implementation of prevention science to eliminate healthcare inequities in cardiovascular health [33].

Community-embedded healthcare models may offer an alternative approach.

Rather than asking communities to access healthcare institutions, community-based prevention initiatives seek to reposition healthcare within environments already embedded in everyday life. Such approaches recognize that accessibility extends beyond physical proximity and additionally includes dimensions of familiarity, trust, cultural context and social participation [16,17].

Mosques represent particularly interesting environments for such initiatives. Beyond their religious role, mosques frequently function as community infrastructures where social interaction, education and intergenerational exchange occur. Their existing social networks may provide opportunities for healthcare engagement among populations potentially insufficiently reached through conventional settings [18].

Importantly, the present initiative was intentionally designed as more than a screening intervention alone.

Detection of elevated blood pressure, cholesterol or glucose values represents only one component of preventive care. Identification of cardiovascular risk factors does not necessarily translate into healthcare engagement or behavioral change. Existing evidence suggests that communication, health literacy and trust strongly influence whether risk identification ultimately results in meaningful health outcomes [10,12,23,34,35].

The integration of educational sessions, informal dialogue with healthcare professionals and structured cardiovascular screening therefore aims to create a broader environment for health engagement rather than isolated risk detection.

An additional strength of this protocol is the incorporation of a structured assessment of access to care, trust in healthcare and health literacy. Community interventions often focus primarily on disease prevalence while providing limited insight into mechanisms underlying healthcare disparities. By combining this questionnaire with the health check, this study aims not only to identify cardiovascular risk profiles but also to improve understanding of barriers influencing preventive healthcare participation.

Insights generated through this approach may contribute to the future development of more inclusive prevention strategies and support the implementation of scalable community-based healthcare infrastructures.

Several challenges should also be acknowledged. Community-based initiatives may be subject to selection bias, as participation remains voluntary and individuals attending health initiatives may differ from non-participants. Community-specific findings may additionally not be directly generalizable across populations and settings [36].

This study has further limitations. It has no comparator and therefore cannot assess effectiveness. Screening results are based on a single, non-fasting community measurement and represent screen-positive findings rather than diagnoses. The health check is offered only to visitors aged 40 to 70 years without known cardiovascular disease or relevant medication, so screening results do not apply to younger or older visitors or to those already under treatment. Previous diagnoses and smoking are not recorded in the questionnaire, so previously unidentified risk factors cannot formally be determined; for this reason, and because external validation of SCORE2 [37,38] in the multi-ethnic HELIUS cohort indicated that its performance differs between ethnic groups [39], an overall risk estimate is not calculated. Educational level, socioeconomic indicators, the country of birth of the parents and preferred language are not recorded either, as the questionnaire was kept short to keep participation low-barrier. Follow-up of abnormal results takes place through the general practitioner, according to the procedure of the Dutch Heart Foundation, and is not part of this study; we therefore cannot determine whether screen-positive findings led to further assessment or treatment. The collection of sociodemographic and healthcare access data allows us to describe which groups are reached.

Nevertheless, community-centered prevention initiatives may provide important opportunities to complement conventional healthcare pathways and improve understanding of populations insufficiently reached through existing preventive systems.

Ultimately, improving cardiovascular prevention may depend not only on the refinement of risk prediction tools or the expansion of screening capacity but also on reconsidering where and how preventive healthcare is delivered.

## 4. Conclusions

This protocol describes a community-embedded cardiovascular prevention initiative that integrates health education, direct interaction with healthcare professionals and low-barrier cardiovascular screening within a trusted community setting. By bringing prevention into an environment where people already gather, the initiative aims to reach populations that are often insufficiently served by conventional preventive care pathways, in line with the inverse care law. Beyond identifying cardiovascular risk factors, the accompanying questionnaire is intended to improve understanding of access to care, trust, health literacy and the barriers that shape engagement with preventive healthcare. The insights gained will inform future implementation and the design of subsequent prevention strategies.

## Figures and Tables

**Table 1:** Eligibility criteria.

Criterion	Description
Inclusion	Visitor of a participating health day Age 18 years or older Written informed consent
Health check	Offered by the Hartcheckpunt to visitors aged 40 to 70 years without known cardiovascular disease and without medication for blood pressure, cholesterol or glucose
Exclusion	Age under 18 years
Notes	No further selection. Bilingual team members and interpreters are present, and the questionnaire is available in Dutch, Turkish, Moroccan Arabic, English, Spanish, Hindi and Punjabi. Participation in the health day, including the health check, does not depend on participation in this study.

**Table 2:** Description of the health day according to TIDieR.

TIDieR Item	Description
1. Name	Mosque-based health day combining education, dialogue with healthcare professionals and a cardiovascular health check
2. Why	Inverse care law: prevention delivered in a trusted community setting may reach people who are less connected to conventional preventive care
3. What (materials)	Education: short slide presentations prepared by the research team, available from the corresponding author on request. Health check: standard equipment of the Hartcheckpunt. Questionnaire: paper, in Dutch, Turkish, Moroccan Arabic, English, Spanish, Hindi and Punjabi
4. What (procedures)	Collective educational session; informal dialogue with healthcare professionals; health check (visitors aged 40 to 70 years) with results and referral advice, followed by general lifestyle advice on elevated risk factors (10 to 15 min); questionnaire for consenting participants
5. Who provided	Education and dialogue: physicians and researchers of the team. Health check, results and referral advice: Hartcheckpunt staff. Lifestyle advice: lifestyle coaches and members of the TRAIN team, at the request of the Dutch Heart Foundation. Registration and logistics: mosque volunteers. Language support: bilingual team members and interpreters
6. How	Face-to-face; education collectively, health check and lifestyle advice individually
7. Where	Mosque Al-Kabir, Amsterdam
8. When and how much	Three to four health days per year; on a Friday afternoon after Friday prayer; collective session approximately 45 min; lifestyle advice 10 to 15 min
9. Tailoring	Setting and timing; announcement by the imam; attention to health within Islamic teaching; language support; culturally relevant examples on food, family life and Ramadan
10. Modifications	Changes between health days are recorded and reported
11. How well (planned)	Fidelity checklist per health day: components delivered, duration, staff and volunteers, language support, deviations
12. How well (actual)	Reported in the Results paper

**Table 3:** Outcomes.

Outcome	Definition	Measurement	Time Point	Analysis
Primary: health perception	Perceived healthiness of own lifestyle	Questionnaire item 10	Health day	% (95% CI)
Primary: access to care	Ease of GP appointment; forgone care and reasons	Items 5 to 7	Health day	% (95% CI)
Primary: trust and experience	Trust in Dutch healthcare; feeling understood by the physician	Items 8 and 9	Health day	% (95% CI)
Primary: health literacy	Limited versus adequate health literacy	Items 13 to 15 (adapted SBSQ)	Health day	% (95% CI)
Secondary: screen-positive risk factors	Hartcheckpunt cut-off values; not a diagnosis	Health check	Health day	% (95% CI)
Secondary: relation with primary outcomes	Association between perception, access, health literacy and screen-positive findings	Questionnaire and health check	Health day	χ^2^, *t*-test or equivalent
Secondary: feasibility	RE-AIM indicators (Section 2.9.3)	Entrance registration, consent and completion records	Health day	% (95% CI), counts
Secondary: information preferences	Topics, setting, preferred channel	Items 11 and 16 to 18	Health day	%

CI, confidence interval; GP, general practitioner;; SBSQ, Set of Brief Screening Questions.

**Table 4:** Schedule of enrollment and assessments.

Activity	Before Health Day	Health Day: Arrival	Health Day: After Health Check
Announcement by imam and mosque	X		
Registration at the entrance		X	
Collective educational session		X	
Health check, results and lifestyle advice		X	
Dialogue with healthcare professionals		X	X
Information and informed consent			X
Questionnaire			X
Recording of referral advice			X
Fidelity checklist		X	X

X, activity takes place at this time point.

## Data Availability

The data presented in this study are available from the corresponding author upon reasonable request, in accordance with the consent obtained and the General Data Protection Regulation.

## Supplementary Materials

The following supporting information can be downloaded at https://www.mdpi.com/article/10.3390/jcdd13100525/s1. Table S1: SPIRIT 2025 checklist, adapted for an observational study; Table S2: TIDieR checklist; File S1: Study questionnaire (English translation); Table S3: Precision of estimated proportions by sample size.
