# Supplementary material for: Bringing Cardiovascular Prevention into the Community: Protocol for an Observational Study of a Mosque-Based Health Initiative Combining Education and Cardiovascular Screening
Source: J Cardiovasc Dev Dis. 2026 Oct 19;13(10):525. doi: 10.3390/jcdd13100525 (PMC13705222; doi:10.3390/jcdd13100525)
Supplement: Supplementary file 1 [file jcdd-13-00525-s001.zip › jcdd-4559318-supplementary.pdf]

Supplementary Materials

Bringing Cardiovascular Prevention into the Community: Protocol for an Observational Study of a Mosque-Based Health Initiative Combining Education and Cardiovascular Screening

Contents: Table S1, SPIRIT 2025 checklist;

Table S2, TIDieR checklist;

File S1, study questionnaire (English translation);

Table S3, precision of estimated proportions by sample size. Locations refer to section numbers of the revised manuscript.

Table S1. SPIRIT 2025 checklist, adapted for an observational study

Item descriptions are abbreviated. Full item wording: Chan AW, et al. SPIRIT 2025 statement. BMJ 2025;389:e081477. Items that apply only to randomized trials are marked as not applicable.

Item	Topic	Location in manuscript / comment
**Administrative information**		
1a	Title	Title page
1b	Structured summary	Abstract
2	Protocol version	Protocol reviewed by the non-WMO review committee, version 3.0, 15 July 2026
3a	Roles and responsibilities: contributors	Author Contributions
3b	Sponsor	Amsterdam UMC (sponsor according to the reviewed protocol)
3c	Role of sponsor and funders	Funding statement; no external funding
3d	Committees	No steering or data monitoring committee; not applicable to this observational study
**Open science**		
4	Registration	Section 2.14 (not registered; observational study)
5	Access to protocol and statistical analysis plan	This publication; Section 2.13
6	Data sharing	Data Availability Statement
7a	Funding	Funding statement
7b	Conflicts of interest	Conflicts of Interest statement
8	Dissemination	Section 2.6 (results shared with the community); publication in scientific journals
**Introduction**		
9a	Background and rationale	Introduction
9b	Choice of comparator	Not applicable (observational study without allocation to an intervention)
10	Objectives	Section 2.1
**Methods: patient and public involvement and design**		
11	Patient and public involvement	Section 2.6
12	Design	Section 2.1
13	Setting	Sections 2.2 and 2.5
14a	Eligibility criteria for participants	Section 2.3; Table 1
14b	Eligibility of sites and of those delivering the activities	Sections 2.5 and 2.7; Table 2
**Methods: activities and outcomes**		
15a	Description of the activities	Section 2.7; Table 2 (the health day is a community activity, not a study intervention)
15b	Criteria for discontinuation or modification	Not applicable (observational study without allocation to an intervention)
15c	Adherence	Not applicable (observational study without allocation to an intervention)

Item	Topic	Location in manuscript / comment
15d	Concomitant care	Sections 2.7.4 and 2.10 (follow-up through the general practitioner, outside the study)
16	Outcomes	Section 2.9; Table 3
17	Harms	Section 2.7.4 (procedure for markedly abnormal results); no study-related harms expected
18	Participant timeline	Section 2.14; Table 4; Figure 1
19	Sample size	Section 2.12; Table S3
20	Recruitment	Section 2.5
**Methods: assignment and blinding**		
21a to 23	Sequence generation, allocation concealment, implementation	Not applicable (observational study without allocation to an intervention)
24a to 24c	Blinding	Not applicable (observational study without allocation to an intervention)
**Methods: data collection, management and analysis**		
25a	Data collection methods	Sections 2.7.3 and 2.8; File S1
25b	Retention and complete follow-up	Not applicable (cross-sectional design); withdrawal procedure in Section 2.4
26	Data management	Section 2.11
27a to 27d	Statistical methods, additional analyses, analysis population and missing data	Section 2.13
**Methods: monitoring**		
28a, 28b	Data monitoring committee and interim analyses	Not applicable (observational study without allocation to an intervention)
29	Trial monitoring	Not applicable; quality control of data in Section 2.11
**Ethics and dissemination**		
30	Ethical review	Section 2.4; Institutional Review Board Statement
31	Protocol amendments	Changes to the protocol are submitted to the non-WMO review committee of Amsterdam UMC
32a	Informed consent	Section 2.4
32b	Additional consent provisions	Section 2.4 (separate consent for use of health check results and for storage of data for future research)
33	Confidentiality	Section 2.11
34	Ancillary care and compensation	Sections 2.7.4 and 2.10; no financial incentives (Section 2.3)

Table S2. TIDieR checklist

Template for Intervention Description and Replication (Hoffmann TC, et al. BMJ 2014;348:g1687), applied to the health day.

Item	Topic	Location in manuscript / comment
1	Brief name	Title; Table 2
2	Why: rationale and theory	Introduction (inverse care law); Table 2; Figure 2
3	What: materials	Section 2.7.1; Table 2; File S1 (questionnaire); presentations available on request
4	What: procedures	Sections 2.7.1 to 2.7.4; Table 2
5	Who provided	Sections 2.5, 2.7.1 and 2.7.3; Table 2
6	How: mode of delivery	Section 2.7.1; Table 2
7	Where	Section 2.2; Table 2
8	When and how much	Sections 2.7.1 and 2.14; Table 2
9	Tailoring	Sections 2.5 and 2.7.1; Table 2
10	Modifications	Table 2 (changes between health days are recorded and reported)
11	How well: planned fidelity assessment	Table 2 (fidelity checklist per health day); Section 2.9.3
12	How well: actual fidelity	To be reported in the results paper

File S1. Study questionnaire (English translation)

Questionnaire: Health and Care (Mosque Health Day), version 2.0, 3 June 2026. Translated from the original Dutch version. The questionnaire is also available in Turkish, Moroccan Arabic, Spanish, Hindi and Punjabi.

Question	Response options
**Background characteristics**	
1. What is your age?	< 30 / 30 to 45 / 45 to 60 / > 60
2. What is your sex?	Male / Female / Other or prefer not to say
3. Were you born in the Netherlands?	Yes / No
4. If not, how long have you lived in the Netherlands?	< 10 years / 10 to 20 years / > 20 years
**Access to care**	
5. How easy is it for you to make an appointment with your general practitioner?	Very easy / Fairly easy / Difficult / Very difficult
6. Have you ever needed care but not sought help?	Yes / No
7. If yes, why? (multiple answers possible)	Language problems / No trust / Shame / No time / Did not know where to go / Other: ...
**Trust and experience**	
8. How much trust do you have in Dutch healthcare?	A lot of trust / Reasonable trust / Little trust / No trust
9. Do you feel understood by your doctor?	Always / Usually / Sometimes / Rarely
**Health and lifestyle**	
10. How healthy do you consider your own lifestyle?	Very healthy / Fairly healthy / Unhealthy
11. Which topics would you like to learn more about? (maximum 3)	Nutrition / Physical activity / Stress / Heart disease / Diabetes / Quitting smoking / Other: ...
12. Have you ever had your blood pressure measured?	Yes / No / Don't know
13. How often do you need help reading information from the doctor or pharmacy?	Never / Rarely / Sometimes / Often / Always
14. How confident are you that you can fill in medical forms yourself?	Very confident / Confident / Somewhat confident / Not confident
15. How often do you have difficulty understanding written information about health?	Never / Rarely / Sometimes / Often / Always
**Community and intervention**	
16. Would you like to receive health information in the mosque?	Yes / No
17. What is the best way for you to receive information?	Lecture / Personal conversation / Leaflet / Video / Via the imam / Other: ...
18. Would you take part in a health check (for example blood pressure, cholesterol)?	Yes / No / Maybe

Table S3. Precision of estimated proportions by sample size

Expected 95% confidence intervals (Jeffreys method) for an observed proportion of 10%, 30% and 50%.

Number of participants	Proportion 10%	Proportion 30%	Proportion 50%
200	6.4% to 14.7%	24.0% to 36.6%	43.1% to 56.9%
400	7.3% to 13.2%	25.7% to 34.6%	45.1% to 54.9%
600	7.8% to 12.6%	26.4% to 33.8%	46.0% to 54.0%
1000	8.3% to 12.0%	27.2% to 32.9%	46.9% to 53.1%
